# In Situ Macroscopic Tensile Testing in SEM and Electron Channeling Contrast Imaging: Pencil Glide Evidenced in a Bulk β-Ti21S Polycrystal

**DOI:** 10.3390/ma12152479

**Published:** 2019-08-04

**Authors:** Meriem Ben Haj Slama, Nabila Maloufi, Julien Guyon, Slim Bahi, Laurent Weiss, Antoine Guitton

**Affiliations:** 1 Université de Lorraine–CNRS–Arts et Métiers ParisTech–LEM3, 7 rue Félix Savart, 57070 Metz, France; 2 Labex Damas–Université de Lorraine, 57073 Metz, France

**Keywords:** in situ, tensile test, plastic deformation, SEM, A-ECCI, slip-system, dislocation, BCC titanium

## Abstract

In this paper, we report the successful combination of macroscopic uniaxial tensile testing of bulk specimen combined with In situ dislocation-scale observations of the evolution of deformation microstructures during loading at several stress states. The dislocation-scale observations were performed by Accurate Electron Channeling Contrast Imaging in order to follow the defects evolution and their interactions with grain boundaries for several regions of interest during macroscopic loading. With this novel in situ procedure, the slip systems governing the deformation in polycrystalline bulk β-Ti21S are tracked during the macroscopic uniaxial tensile test. For instance, curved slip lines that are associated with “pencil glide” phenomenon and tangled dislocation networks are evidenced.

## 1. Introduction

Developing innovative techniques that allow for understanding and predicting the mechanical properties of materials has become a necessity for the Materials Science community, in order to follow the fast evolution of the industrial demands [1,2,3].

Generally, mechanical testing is preceded and/or followed by microstructural investigations in order to get the structure-property-processing relationships [4,5,6,7]. In situ characterization provides more useful data for a more realistic theoretical modeling, which allows for predicting the mechanical performance of components. In situ mechanical testing in Transmission Electron Microscope (TEM) [8,9,10] is well known for studying the evolution of crystalline defects under stress. Various approaches of mechanical testing inside a TEM exist: using TEM holders with a simple mechanical actuation [11] or elaborating testing-units that fit inside the pole pieces [12,13]. While TEM allows reaching the highest-resolution data, In situ TEM mechanical testing is experimentally difficult and it does not necessarily reflect the macroscopic response of a material. In addition to the difficult control of the deformation conditions, the space that is available inside the microscope chamber is generally less than one centimeter and statistics on physical mechanisms are low. Indeed, TEM needs an electron transparent specimen with a thickness of ≈ 100 nm with a useful field of view of few µm^2^. Therefore, fundamental questions regarding the representativeness of observed phenomena must be raised when extrapolating discussions to a millimeter-size specimen or higher (centimeter scale, macroscopic testing).

In Scanning Electron Microscope (SEM), in situ mechanical testing is also used for studying the evolution of microstructures during exposure to stress. For instance, in situ Electron BackScatter Diffraction (EBSD) is performed in several studies to follow the deformation of materials, such as aluminum alloys during tensile tests [14,15].

In addition to EBSD analysis, direct SEM observations have been reported while using Electron Channeling Contrast Imaging (ECCI) to characterize for example, cracks in metals, such as NiAl single crystal [16] or also *post mortem* observation of subgrain-boundaries structures formed in ceramics during deformation [5,17,18].

In the work that is described in this paper, we successfully carried out Accurate ECCI (A-ECCI) characterization during the macro-mechanical testing of a polycrystalline bulk titanium alloy specimen. With A-ECCI, the TEM extinction criteria can be applied on bulk samples [19,20,21,22,23]. For a precise analysis of defects, it is mandatory to accurately control the relative orientation of the crystal to the incident electron beam (i.e., optical axis of the SEM), because of its strong effect on the BackScattered Electrons (BSE) yield. This is achieved through the A-ECCI procedure. The precise orientation of the crystal in the SEM coordinate system is given through High-Resolution Selected Area Channeling Patterns (HR-SACP) that were developed some years ago [23]. It is obtained by rocking the incident electron beam at a pivot point on the surface of the sample [23]. HR- SACP allows for an angular accuracy better than 0.1° with a spatial resolution of less than 500 nm [22].

As a proof-of-principle of the in situ test, the chosen material was β-21S titanium alloy (Ti-15Mo-2.7Nb-3Al-0.2Si). β-titanium alloys, and particularly the β-21S, are good candidates for several applications, such as biomedical [24] and aerospace industries [25], because of a combination of promising mechanical properties. They offer a good fatigue resistance, a wide range of strength to weight ratios, a deep hardening potential, and an inherent ductility that is promoted by their Body-Centered Cubic (BCC) structure [26,27,28,29]. In addition, β-21S titanium exhibits high cold-formability. When plastically deformed at room temperature, its initial equiaxed grain morphology does not change, irrespective of the testing direction [30]. Many slip bands aligned at ~35–50° to the axis of tensile loading develop within the grains; the deformation is governed by a dominant dislocation/slip mechanism [30]. The outcomes of our experimental setup open the way to the microstructure evolution study during deformation, especially by A-ECCI for the precise characterization of crystalline defects during macroscopic testing.

## 2. Materials and Methods

Bulk tensile samples were cut from a 1.78 mm thickness rolled sheet of β-Ti21S alloy, which were produced by Titanium Metals Corporation (Toronto, ON, Canada). The exact chemical composition of the material is Ti-15.97Mo-2.79Nb-2.99Al-0.26Fe-0.2Si. As a heat treatment, a soaking at 843 °C for 14 min. was first applied before air cooling. The tensile samples were cut parallel to the rolling direction by using waterjet cutting (garnet 80 mesh, 3500 bar) to avoid heating and microstructure changing.

The tensile tests were performed at room temperature with a DEBEN (Suffolk, UK) machine of a maximum load cell of 1 kN. The measurements were taken under an imposed force and with a strain rate of 3.3 × 10^−4^ s^−1^.

For EBSD and A-ECCI characterization experiments, the sample was mechanically polished with 1 µm diamond paste, followed by chemo-mechanical polishing with colloidal silica suspension in order to produce a very flat surface and minimize any work hardening, due to conventional grinding. Finally, 2 h ion-polishing in a PECS II (GATAN, Pleasanton, CA, USA) machine was applied with a 3 keV beam, to improve the surface quality, so that a higher signal to noise ratio can be obtained for the BSE signal. 

Detailed in situ characterizations of the microstructure during deformation were performed by A-ECCI while using a Zeiss Auriga Scanning Electron Microscope (SEM, Oberkochen, Germany) operating here at 10 kV. Primarily to this step, to get the approximate grains orientation in the microscope coordinate, EBSD experiments were carried out, before deformation, in a Zeiss Supra 40 SEM (Oberkochen, Germany) that was operating at 20 kV. This necessary step allows for superimposing the HR-SACP of low angular range of 4.4°, on an EBSD pattern that was simulated at 0°, justified by the ECCI experiments at low tilt.

## 3. Results

**Figure 1:**
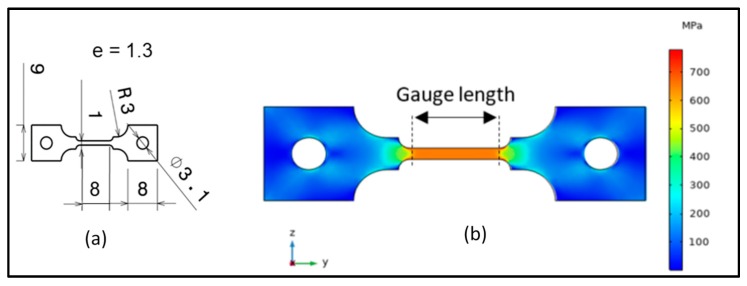
(**a**) Tensile sample geometry and sizes (mm); (**b**) Elastic deformation simulation on COMSOL.

Figure 1a shows the geometry and the sizes of the tensile sample, which were designed to fit in the tensile machine.

A simulation of the elastic regime was first performed by COMSOL in order to confirm that the stress field is uniaxial and homogenous along the operational length of the sample [31]. It was done while using a free tetrahedral meshing applying elements of 0.6 to 3.4 mm size. The Young modulus used was an averaged value of β-Ti21S Young modulus found in literature; around 81 GPa [32] to 85 GPa [33]. This simulation shows that the stress field is homogenous and it exhibits no stress concentration zones in the gauge length.

**Figure 2:**
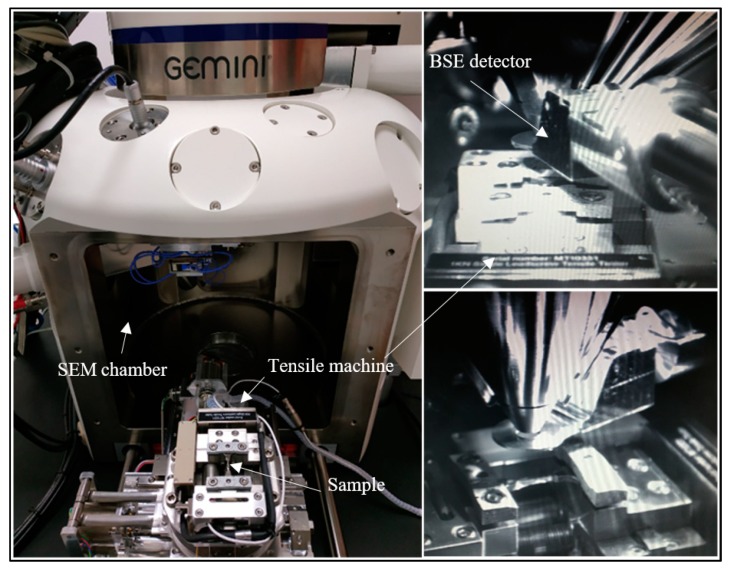
Assembly of the tensile machine/sample inside the Scanning Electron Microscope (SEM); when SEM chamber is opened (left image) and inside the closed SEM chamber with the BackScattered Electrons (BSE) detector inserted (right images).

Figure 2 shows the assembly of the tensile machine and the sample inside the SEM. The set-up allows for a tilt of about 15° inside the SEM chamber, with the BSE detector being inserted.

**Figure 3:**
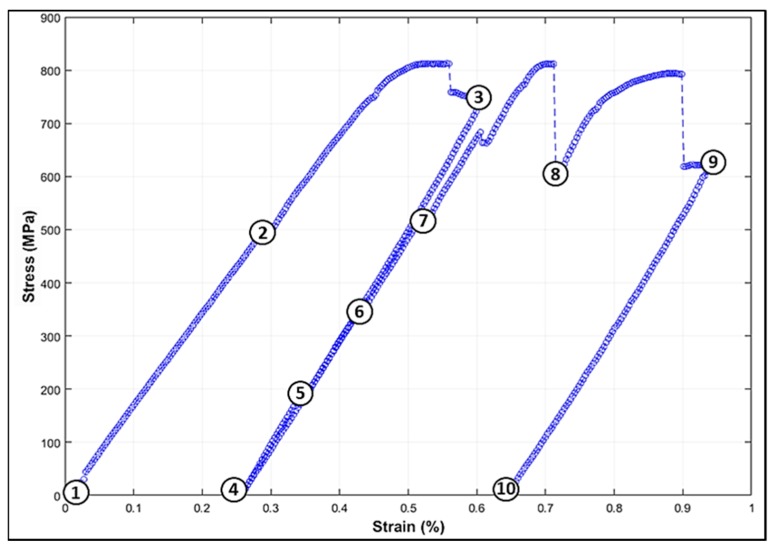
In situ obtained stress-strain curve of the β-Ti21S showing the positions where observations/Accurate Electron Channeling Contrast Imaging (A-ECCI) were made: before loading (1), during elastic deformation (2: no defect formation was observed), at the beginning of the plastic domain (3), and for six intermediate states of the second tensile cycle before unloading (four to 10). The dashed vertical lines correspond to the relaxation after plastic deformation.

In situ tensile tests and SEM observations were performed, as seen in Figure 3, with the following three steps:

- Step 1: An observation (+imaging) of a maximum number of the sample areas before any loading (1 on the curve).
- Step 2: A preliminary load, up to the macroscopic yield stress, to determine the different areas of interest where deformation mechanisms are activated (seven zones were selected to be investigated under further stress states) (two and three on the curve).
- Step 3: A second cycle, which was paused at intermediate stress states to carry out A-ECC imaging of each area of interest (four to 10 on the curve). The characterization was made after 15 min. of relaxation (dashed vertical lines on the curve).

## 4. Proof-Of-Principle and Discussion

In this paper, we report the first results illustrating the potential of in situ experiments coupled with A-ECCI without going further in analyzing the observed deformation mechanisms. Deeper and more detailed studies of the highlighted phenomena will be done in the incoming papers.

**Figure 4:**
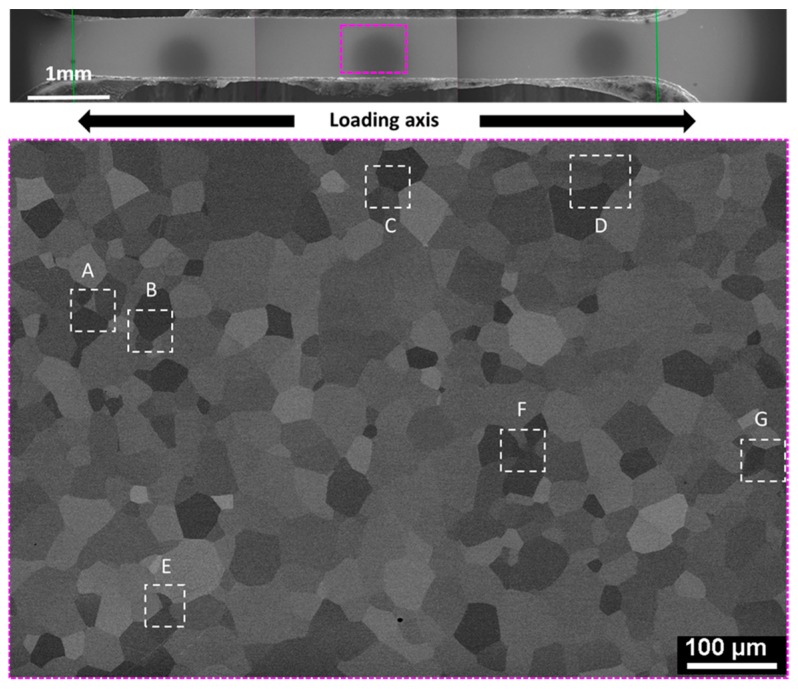
Observed area of 600 × 450 µm^2^ with the seven zones of interest in white dashed frames.

The observed area was chosen to be in the middle of the sample in a way to cover almost the whole width. It is also chosen far away from the edges in order to avoid any edge effects. Seven zones showing several configurations of slip-traces/dislocations are identified, as seen in Figure 4. 

The channeling conditions were chosen to optimize the defect contrast, as detailed in the introduction section and in the above-mentioned references [19,20,21,22,23].

Briefly, it is reported that better contrast is obtained when the incident beam is oriented near a pseudo-Kikuchi band edge, where the BSE yield is minimal. This corresponds to a channeling condition that is associated with the darkest contrast of the grain [34]. For each of the 7 selected zones, the channeling contrast was optimized in order to obtain a simultaneous good channeling condition on both sides of the grain boundary.

**Figure 5:**
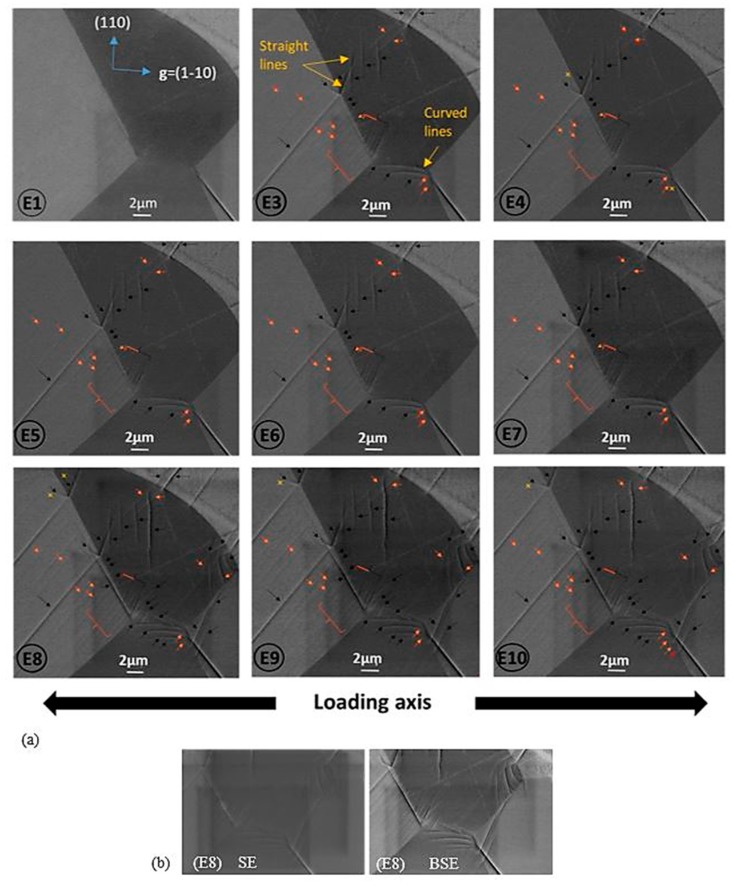
(**a**) BSE micrographs in channeling condition (with **g** = (1–10) in the grain of interest) presenting the evolution of the defect structure corresponding to each deformation step of the tensile curve. The letter refers to the zone (E) and the number refers to the load state from the curve. Black arrows indicate slip lines, orange arrows indicate dislocations and yellow crosses indicate an increase in dislocation length; (**b**) Example of SE vs BSE images from zone (E8).

Given this working conditions, the diffraction vector **g** = (1–10) (see example of Figure 5) was chosen to enhance the defects contrast.

We report observations for the area E, chosen because it contains several deformation features, since the paper objective is mainly to explore the potential of the in situ experiment. Figure 5 shows the evolution of the defect structure associated with the several load states for the “E” zone; the letter refers to the zone (E) and the number refers to the load state from the curve. No defect is observed before loading. When plastically deformed, several slip systems are activated and dislocations appear, all at once. 

Dislocations are easily distinguished from surface slip traces, because their contrast is not visible on the obtained images with the Secondary Electron (SE) detector, while they are visible with the BSE detector (if **g**·**b**≠0, where **b** is the Burgers vector) (see Figure 5b).

Concerning the slip lines, we notice the presence of several configurations: straight slip lines appearing in the middle of the grain, straight slip lines nucleating at the grain boundaries and developing in both grains and curved slip lines starting from the grain boundary. 

Concerning dislocations, some of them are arranged in parallel straight lines: they seem to form future slip lines if the sample is more and sufficiently deformed. Other dislocations appeared individually next to slip lines, or in the line continuity. These ones are longer at the unloaded state (E4).

**Figure 6:**
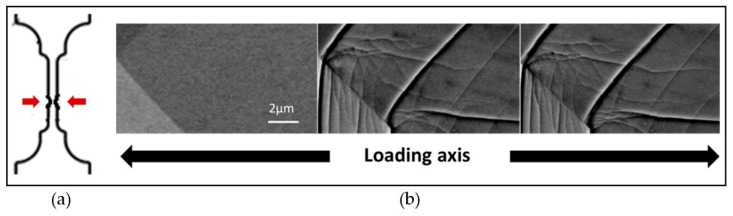
(**a**) Local section reduction to create high-strained zones (**b**) BSE micrographs in channeling condition *post mortem* test showing dislocation networks and slip lines forming and evolving during plastic deformation.

In other cases, the loading induced much more complicated deformation structures, like the one that is shown in Figure 6 where dislocation networks interacting with the grain boundary are observed. The latter structure was obtained from a *post-mortem* tensile test used also as a proof-of-concept, and where high-strained zones were created by a local section reduction.

We encountered particularly configurations showing curved slip-lines among the studied deformation structures during the β-Ti21S tensile test. These lines can be explained by a “pencil glide” mechanism, which is characteristic of the BCC structure, and generally observed in iron [35]. In BCC, {110}, {112}, and {123} planes have comparable atomic packing densities and they contain <111> directions. Therefore, <111>-dislocations can slip into each of these gliding planes [36]. The reasons why one plane is preferred over the other is still unclear. It turns out that dislocations can easily change their slip planes during the deformation. Thus, the slip traces on the surface result in zigzag [37,38].

**Figure 7:**
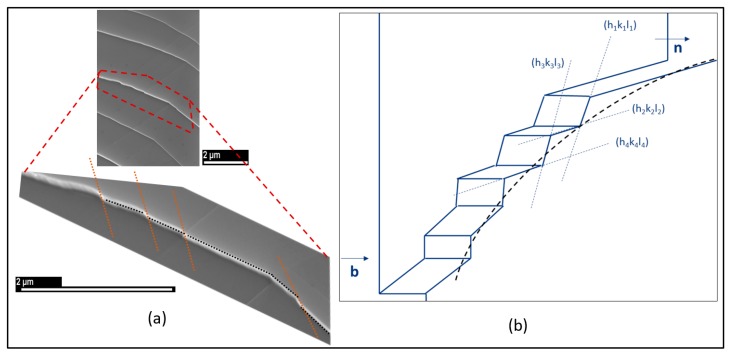
(**a**) Secondary Electron (SE) micrographs showing curved slip lines resulting from pencil glide phenomenon and (**b**) a corresponding schematic representation.

Figure 7a is a SE micrograph of the slip “curves” in β-Ti21S. The schematic representation of Figure 7b corresponds to the slip curve zoomed in Figure 7a. The dislocations move from a slip plane (h_i_k_i_l_i_) to another (h_j_k_j_l_j_) forming a stair-like shape, which results in a curved appearance when the images are taken at a lower magnification.

These preliminary results open the path for deeper investigations of dislocation/slip systems activated during deformation and their characterization. The described in situ approach will allow a better understanding of fundamental deformation mechanisms, such as pencil glide in BCC crystalline structures, or slip transfer through grain boundaries. These mechanisms will be described more in detail in the incoming publications.

## 5. Conclusions

In this paper, we successfully combined, in situ macroscopic tensile testing of a bulk polycrystalline specimen with microstructure observations inside a SEM under controlled channeling conditions. For the first time, using A-ECCI, we evidenced the defect evolutions under stress in a β-21S titanium alloy (Ti-15Mo-2.7Nb-3Al-0.2Si), illustrating the potential of in situ experiments coupled with A-ECCI.

This experiment allows for covering large areas for study, which offers statistically relevant multiscale information to deepen the deformation mechanisms. Allowing a freedom of tilt up to 15°, our set up gives the possibility to comprehensively characterize defects on a bulk specimen by applying the TEM extinction criteria.

The observed slip line/dislocation configurations present an interesting track to explore and determine the involved mechanisms.
